# Magnetic resonance acoustic radiation force (impulse) imaging (MR-ARFI)

**DOI:** 10.1186/2050-5736-3-S1-O34

**Published:** 2015-06-30

**Authors:** Kim Butts Pauly

**Affiliations:** 1 Stanford University, Stanford, CA, United States

## Background/introduction

MR-ARFI adds to the rich toolbox of MR imaging methods for guiding focused ultrasound. MRI has exquisite soft tissue contrast for targeting focused ultrasound and a variety of contrast mechanisms for assessing the effect of focused ultrasound. In addition, MR can provide temperature mapping in near-realtime. MR-ARFI is an imaging method that compliments these other capabilities: MR-ARFI can provide a very low temperature rise method to locate the focal spot, calibrate the beam intensity, and potentially evaluate and improve focusing. MR-ARFI typically uses a relatively long ultrasound pulse (1-20 ms) during the application of a magnetic field gradient to encode the displacement of tissue into the phase of the MR image. MR-ARFI is related to elastography, shear-wave imaging, and harmonic motion imaging methods, which evaluate tissue stiffness by evaluation of the ultrasound shear wave that propagates after the ultrasound pulse.

However, MR-ARFI is instead visualizing the quasi-static displacement of tissue during the ultrasound pulse, rather than the shear wave after the pulse.

## Methods

There is no single method for MR-ARFI in either the encoding gradients or the readout method. For the encoding method, early publications used unipolar gradients, similar to diffusion gradients. Inverted bipolar gradients were used to reduce the diffusion weighting, and repeated bipolar gradients to additionally reduce eddy current errors and motion sensitivity. For gradient echo imaging, single bipolar gradients have been used. Many different readout methods have been used including line scan, spin echo, gradient echo, EPI, RS-flyback-SE-EPI, GRE-EPI, 3D, single shot FSE, and fluctuating equilibrium MR.

## Results and conclusions

Applications: For the first two goals, the phase of the MR-ARFI picture has been shown to increase linearly with ultrasound power in phantoms and tissues and *in vivo* and to reasonably agree with ultrasound ARFI *in vivo*. In addition, the focal spot spatial location has been shown *in vivo* to be in good agreement with the location of the subsequent temperature rise. For the third goal of evaluating and improving focusing, there have been several proposed methods. One method for adaptive focusing uses an iterative method that chooses the phase of the elements to maximize the phase of the ARFI image. A second method uses direct inversion of spatially coded emissions. A third method uses simulation to iteratively find the phase aberrations consistent with a single measured MR-ARFI image. While the former two methods require significant time be spent on imaging (with associated ultrasound power deposition), the last method instead spends time on the computation, without the same power deposition. MR-ARFI has also been used to identify calcifications, and evaluate tissue stiffness after FUS.

Discussion: With respect to safety, keeping the amplitude of the ultrasound pulse low reduces heat and possibility of cavitation. Several studies with simultaneous MR-ARFI and temperature imaging have shown that the temperature at the focal spot can rise during an MR-ARFI acquisition, but also that the temperature can remain low with suitable choice of parameters.

